# Study of the Precise Determination of Pipeline Geometries Using UAV Scanning Compared to Terrestrial Scanning, Aerial Scanning and UAV Photogrammetry

**DOI:** 10.3390/s23198257

**Published:** 2023-10-05

**Authors:** Grzegorz Lenda, Natalia Borowiec, Urszula Marmol

**Affiliations:** Faculty of Geo-Data Science, Geodesy, and Environmental Engineering, AGH University of Science and Technology, 30-059 Krakow, Poland; nboro@agh.edu.pl (N.B.); entice@agh.edu.pl (U.M.)

**Keywords:** terrestrial laser scanning, airborne laser scanning, UAV laser scanning, structure from motion, pipelines

## Abstract

Transmission pipelines belong to technical infrastructure, the condition of which is subject to periodic monitoring. The research was to verify whether aerial measurement methods, especially UAV laser scanning, could determine the geometric shape of pipelines with a precision similar to that of terrestrial scanning, adopted as a reference method. The test object was a section of a district heating pipeline with two types of surfaces: matte and glossy. The pipeline was measured using four methods: terrestrial scanning, airborne scanning, UAV scanning and the structure from motion method. Then, based on the reference terrestrial scanning data, pipeline models were created, with which all methods were compared. The comparison made it possible to find that only the UAV scanning yielded results consistent with those of the terrestrial scanning for all the pipes. The differences usually did not exceed 10 mm, sometimes reaching 20 mm. The structure from motion method yielded unstable results. For the old, matte pipes, the results were similar to those of the UAV scan; however, for the new, shiny pipes, the differences were up to 60 mm.

## 1. Introduction

Transmission pipelines are elongated objects that require appropriately accurate but also economical measurement methods. High accuracy, which allows for the study of the detailed geometry of pipelines, can be achieved by terrestrial laser scanning (TLS). However, due to the labour consumption, this method is rather used for short sections. Aerial methods, such as airborne laser scanning (ALS) or photogrammetry from a drone (SfM—Structure from Motion), can be much more effective for measuring long sections. These methods have completely different characteristics: airborne laser scanning has a much lower density of observations, and the accuracy of determining the height is usually a dozen or more centimetres. It is therefore a method that can be used mainly for the approximate location of pipelines in space. The SfM method can generate high-density clouds with a several-centimetres-high accuracy. However, this method is susceptible to lighting conditions as well as reflections and can generate significant measurement errors. In practice, it can be useful mainly for locating pipelines in space and, in good measurement conditions, also for determining their geometry. Scanning drones (ULS—unmanned laser scanning) have been available for several years. These are devices of a very different accuracy that depends on the class of positioning systems installed on board (GNSS, INS) as well as the scanner.

The aim of the research was to determine the precision of ULS measurements in determining the geometry of pipelines compared to other methods: TLS, ALS and SfM. If the results turned out to be stable and comparable to terrestrial laser scanning treated as a reference method, it would mean that ULS could be used not only to locate pipelines in space but also to determine their local deformations.

The reference data were the TLS measurement, based on which the pipeline models were created. They were then used to determine the deviations in the point clouds from all the methods. Deviations were presented in a graphical form that allowed for the visualisation of their values and places of occurrence. Thus, it was possible to observe differences in the geometric shape of pipelines determined using each method.

## 2. Literature Review

The pipeline system plays an important role in various fields of the economy. Each pipeline is affected by numerous factors leading to varying deformations that can be detected as global or local displacements. These displacements are measured using various technologies. One of them is terrestrial laser scanning, which allows one to survey the object with a high accuracy [1,2,3]. The redundancy of TLS scanning data makes it a reliable source of information for deformation analyses, but on the other hand, due to the large spatial extent of the objects, it is both uneconomical and time-consuming [4]. Terrestrial laser scanning has been the subject of numerous studies [5,6,7], in which factors affecting its accuracy have been identified, and methods of their elimination have been determined. A review of the literature [8,9,10,11,12] demonstrates that TLS allows for the measurement of objects with a several-millimetres accuracy.

Due to the large spatial extent of the pipelines, it will be much more effective to use data captured from a low altitude such as data obtained through UAV photogrammetry. Photogrammetry drones are used for applications with lower accuracy requirements, e.g., those related to agriculture [13,14] or topography [15,16], as well as for the analysis of building structures with higher accuracy requirements [17]. In [18], a photogrammetric UAV was used to measure a dam, and the consistency of the results compared to terrestrial scanning was about 3 cm. UAV photogrammetry was also used to measure land deformation [19], where a 1–5 cm accuracy was obtained. Several-centimetres accuracies were also obtained in research studies [20,21] related to archaeology and historical monuments. Research in [22] demonstrates that the use of aerial photographs taken at low altitudes enables obtaining precise and high-resolution products with an accuracy that meets the requirements of mining documentation. The accuracy of photogrammetric methods is mainly conditioned by the height of the flight and the related size of the GSD—Ground Sample Distance. The final studies adopted the terrain accuracy at the level of 1–2 GSD and the vertical accuracy at the level of 2–3 GSD [23]. A very important issue is the proper distribution of GCPs (Ground Control Points) in the surveyed area [24]. Improper configuration of GCPs may cause incorrect determination of the elements of external orientation of the photographs, and thus introduce errors in the final alignment of their block [25]. Due to the low stability of the camera parameters [25,26] and the UAV specificity, the alignment is most often carried out using the self-calibration method. Research studies have demonstrated that the condition for the correct performance of this process is the use of an evenly distributed set of GCPs with known coordinates measured with a high accuracy. Moreover, in the case of using multi-rotor UAVs, the quality of the process can be improved by adding oblique photographs to the configuration [27,28], and in the case of using fixed-wing aircraft, by performing additional transverse flights at different heights [29,30]. This makes it possible to reduce the correlation between the elements of internal and external orientations, and to avoid deformations in the final product, which is the digital terrain model [31]. It should also be remembered that photogrammetric measurements may have variable accuracy depending on the type of surface, object illumination, contrast, reflections, shadows, etc. The accuracy of TLS and photogrammetric UAV measurements can be comparable in good conditions [32,33,34] without the influence of the above factors. Since it is not always possible to eliminate adverse factors, the results of the studies may vary [35]. For example, [36] demonstrates that a polarising filter can effectively reduce reflections when measuring small-scale structures. However, in [37], despite the use of a polarising filter, the measurement of the shiny part of the observatory’s dome was not as accurate as its matte part. An appropriate approach in many research problems is to integrate UAV photogrammetric and terrestrial scanning data. The integration performed for the industrial chimney [38] in the places of full mutual cloud coverage from both methods allowed for measurement compliance at the level of about 10 mm, decreasing to about 30 mm in places of partial mutual cloud coverage. Similar results in terms of accuracy were obtained using both methods for bridge surveys [32].

Recent years have seen the dynamic development of scanning drones. They have been used in environmental research [39,40] and geohazard monitoring [41,42]. When it comes to engineering applications, the potential of scanning drones is used, e.g., to monitor bridge deformation. In [43], potential locations of surface defects, such as cracks, were determined based on lidar data from the UAV altitude. Those studies were continued in [44], where a flight planning method was proposed that took into account the significance level of different areas of the bridge in order to obtain more accurate data. Scanning drones are also used in railway infrastructure surveys. Publication [45] presents a self-adapting algorithm for extraction of rails, wires and masts based on their specific geometric features in the UAV lidar cloud. Some research works are devoted to the use of UAV lidar for monitoring high-voltage lines [46,47]. A probabilistic approach to pipe detection and tracking based on ULS data is proposed in [48]. The method has been tested on both simulation and real data. Pipes are one of the most common objects in an industrial environment; therefore, cylindrical sensing systems provide valuable information for the navigation of remote-controlled robots in industrial applications. When considering the accuracy of scanning drones, the GNSS system is most frequently indicated as the source of errors. In [49], discrepancies of a few centimetres were observed during several flights of a cheap drone. The authors of [50] proposed to adjust the flight trajectory in order to take into account the inaccuracies of navigation systems, using a model based on spline functions. In [51], the accuracy of the RIEGL miniVUX-SYS system was tested on a specially designed test field, which yielded results with a precision of 12 mm. A comparison with a reference terrestrial scanning revealed that the accuracy varied from 20 to 40 mm depending on the direction. Neither Ground Control Points (GCPs) nor trajectory adjustments were included in this study. Similar results were obtained in [52], where a UAV system based on the RIEGL VQ-840-G topographic and bathymetric scanner was tested, achieving a precision of 10 mm for land targets and an accuracy of 20–30 mm. During accuracy tests of the DJI Matrice 300 drone equipped with the Zenmuse L1 scanner [53], errors of about 70 mm were observed, which were reduced to about 35 mm after correcting georeferencing errors. The authors of the research paper [54] even achieved a several-millimetres accuracy using hybrid georeferencing, combining scanning and photogrammetric measurements on a UAV platform. This method was used to determine the deformation of the river lock.

On the basis of the literature review, it can be concluded that the photogrammetric measurements of building and technical objects using drones have an average accuracy of a few centimetres (usually about 3–5 cm). When complemented by data from other measurement methods, SfM measurements can reach an accuracy of up to a dozen millimetres. However, under difficult measurement conditions, the accuracy of this method is variable. Depending on the type of surface, the structure lighting and its background, contrast, reflections, shadows, etc., it can decrease significantly by up to several tens of centimetres. The accuracy of scanning drone surveying (ULS) is currently under investigation and is estimated to be approximately 2–4 cm. An emerging work indicates that, when integrated with data from other measurement methods, this accuracy can be as high as a sub-centimetre level. Additionally, scanning is much less sensitive to factors that disturb the accuracy of photogrammetric measurements. Overall, the SfM method’s application range is now much better documented than the relatively new ULS method.

## 3. Description of the Experiment

**Figure 1:**
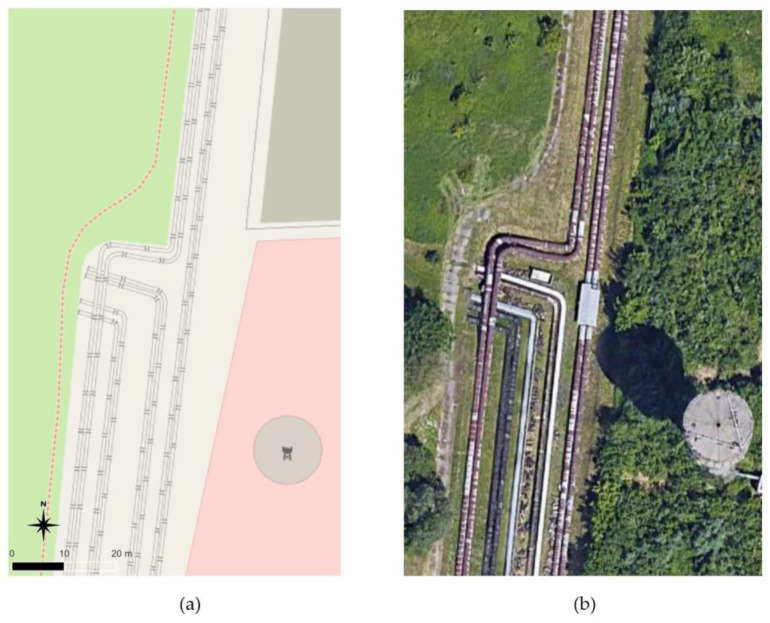
Study area: (**a**) OpenStreetMap, (**b**) orthophotomap.

The research was to verify whether aerial measurement methods, especially UAV laser scanning, could determine the geometric shape of pipelines with a precision similar to that of terrestrial scanning, adopted as a reference method. The test field was a fragment of the heating pipeline located at the Krakow Heat and Power Plant (Figure 1). One of the objectives of the research was to determine how the type of material from which the pipeline was made would affect the accuracy of the study. Therefore, two fragments with different characteristics were selected: old, rusty pipes and new pipes with a shiny surface. The study area covered a section of pipelines about 115 m long. The pipelines were characterised by the same shape, regardless of their length. The results were affected by the measurement method and the type of material covering the pipelines. Half of the tested pipelines were covered with old, rusty sheets, and half with new and shiny sheets.

The object was measured using four methods: reference terrestrial scanning, airborne laser scanning, UAV scanning and the SfM method, all described in detail in the following subsections. The accuracy of aerial methods is dependent on on-board navigational systems. The height-related Z coordinate is usually the least precisely determined. In order to be able to compare the geometry of pipelines from all the methods, it was necessary to minimise the factor related to the accuracy of each method and leave the factor related to the measurement precision. For this purpose, each aerial method was registered with the terrestrial measurement using the cloud-to-cloud method (ICP—Iterative Closest Point). The ICP algorithm minimises the distances between point clouds. The moving cloud is matched to the reference point cloud during successive iterations. During the matching, a transformation matrix containing rotation and translation is determined such that the distances between the points of the clouds reach a minimum. Thus, all the clouds were in the closest possible vicinity. Then, pipeline models were created based on terrestrial scanning data, and spatial charts of deviations of each point cloud from the models were determined. As a result, it was possible to observe differences in the geometric shape of the pipelines determined by each method. The detailed research procedure, which consisted of the following elements, is described below:

- TLS data processing
- ULS data processing
- ALS data processing
- SfM data processing
- registration of ALS, ULS and SfM clouds with the reference TLS cloud
- creating pipe models from TLS data
- analyses of cloud deviations from pipe models

## 4. TLS, ULS, ALS, SfM Measurements

### 4.1. Terrestrial Laser Scanning Data

**Figure 2:**
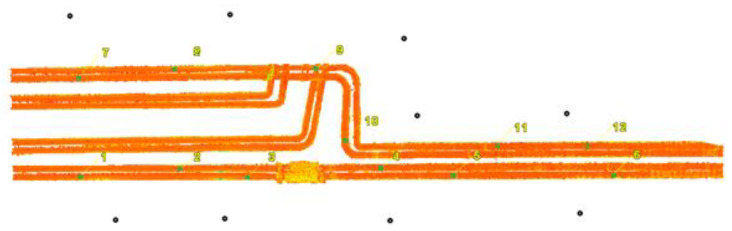
Distribution of measurement stations (black circles) and spherical targets (green rectangles marked with numbers) during terrestrial laser scanning.

The measurement using terrestrial laser scanning was carried out from nine stations located at a mutual distance of approx. 20–40 m (Figure 2). The close proximity of the measurement stations resulted from the need to ensure the appropriate density of points on the pipes. The distances between the points were about 10–30 mm. The measurement was performed using a Leica ScanStation C10 scanner with a measurement accuracy of 6 mm and a precision of 2 mm. The point clouds were merged using twelve spherical targets distributed on the pipeline so that 5–6 targets could be used to merge each pair of stations. Cloud registration was carried out in the Leica Cyclone v.2023.0.2 software. The average registration error was 2 mm, and the errors on individual targets reached 1–4 mm.

**Figure 3:**
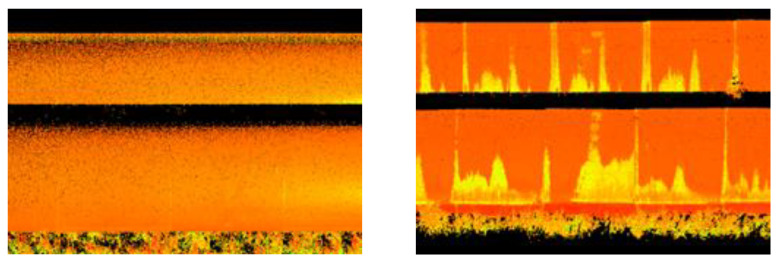
Fragments of the pipeline measured by terrestrial laser scanning: **left**—new pipes (shiny), **right**—old pipes (matte).

Figure 3 illustrates examples of sections of the new and old pipelines measured by terrestrial laser scanning. Differences in beam reflection intensity make it easy to distinguish between old and new pipes.

### 4.2. UAV Scanning Data

**Figure 4:**
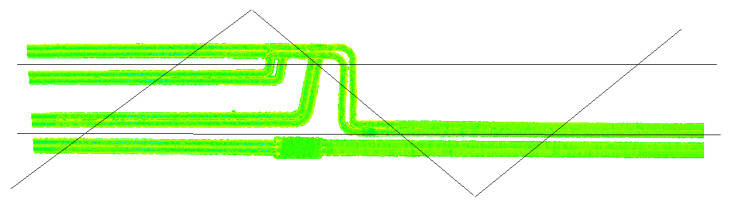
Trajectories of the scanning drone: two longitudinal ones and a zigzag one.

Lidar data were captured during a UAV flight, where a Riegl Ricopter drone equipped with a VUX-1UAV scanner was used to measure the point cloud. The accuracy of the scanner was 10 mm, the precision was 5 mm, and the scanning speed was 500,000 points per second. The drone had an IMU (Inertial Measurement Unit) and a Trimble AP-20 GNSS (Global Navigation Satellite System) unit. Two flight trajectories along the pipelines and one zigzag trajectory were planned in order to better visualise the side surfaces of the pipelines (Figure 4). The drone flew at a speed of 6 m/s at an altitude of approx. 30 m above the pipeline.

**Figure 5:**
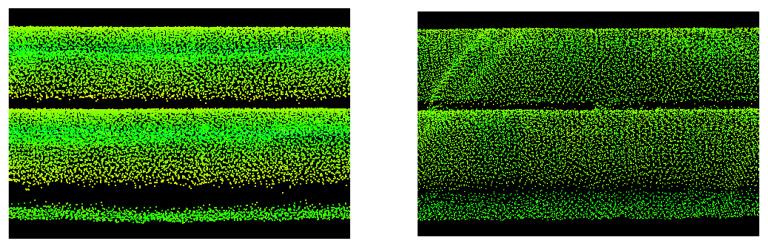
Fragments of the pipelines measured with UAV laser scanning: **left**—new pipes (shiny), **right**—old pipes (matte).

Using dedicated software from the manufacturer Riegl Pospac, flight trajectories were determined. Average trajectory adjustments were 15.5 mm for positions and 0.011 degrees for angles. Then, point clouds from individual trajectories were determined and registered in the Riprecision v.1.9.2. software. The uniform point cloud had noticeable, several-centimetres, systematic shifts between the clouds from different trajectories. Therefore, the registration in the Trimble Realworks v.12.2 software, which had more configuration possibilities, was repeated. The individual trajectories were combined using the cloud-to-cloud method (ICP—iterative closest point), and the average registration error was 23 mm. The density of the ULS measurement was lower than that of the TLS measurement. The distances between the points of the ULS cloud were between 20 and 50 mm. Figure 5 illustrates exemplary fragments of the new and old pipelines measured by UAV laser scanning. The intensity of the beam reflection from old and new pipes exhibited only slight differences.

### 4.3. Airborne Laser Scanning Data

The airborne laser scanning data came from the ISOK project [55]. ISOK is an IT system for country protection against extreme hazards; it is aimed at protecting the economy, the environment and the society against disasters, primarily against floods. The ISOK project is co-financed by the European Regional Development Fund as a part of the Innovative Economy Operational Program—Priority Axis 7. The point cloud obtained in this project for the selected area has a density of 12 points/m², with an average distance between points of about 0.3 m. The analysed area is located in the map section denoted with the number 7.125.12.11 of the PL-2000 coordinate system at the 1:2000 scale. Elevation data include additional information such as intensity, classification and echoes, which can be a significant source of information in cloud processing. According to the research carried out in [56], the absolute height accuracy for the data from the ISOK project varies for particular types of land cover from slightly more than 10 cm for hardened surfaces to more than twice as high for forest areas.

**Figure 6:**
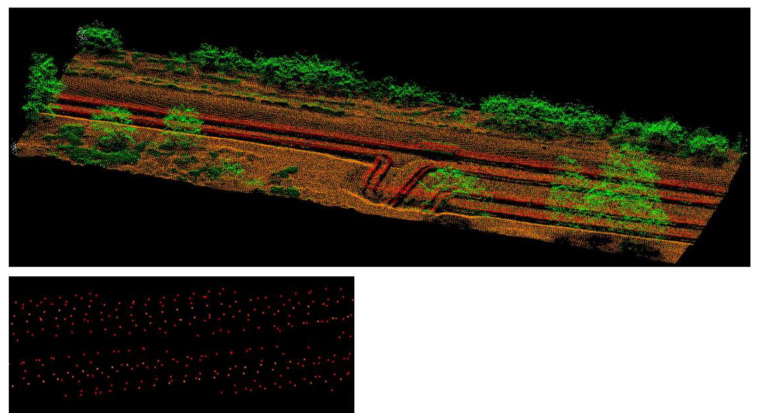
Visualisation of data from ALS cropped to the study area, together with an enlarged fragment of the pipeline.

Figure 6 demonstrates the point cloud obtained from airborne laser scanning for the entire area and an enlarged example of a fragment of the pipeline. The reflection intensity for old pipes and new pipes is indistinguishable. The low density of points means that the clouds do not create an image of pipes with a compact outline.

### 4.4. UAV Photogrammetric Data

**Figure 7:**
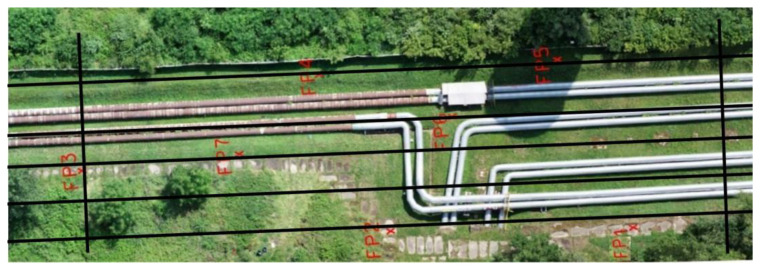
Orthophotos of the analysed area with measured GCPs and marked UAV flightline.

Photogrammetric data were taken during the DJI Phantom 4 Pro unmanned aerial vehicle’s flight. During the photogrammetric mission, 812 images were taken, which were arranged in 5 longitudinal and 2 transverse flightlines located on the edges of the studied area (Figure 7). The coverage between the images was about 80%. The average GSD of the acquired images was 0.8 cm. In order to spatially locate the captured data, seven GCPs were measured in the field using the GPS technique in the 2000PL system.

**Figure 8:**
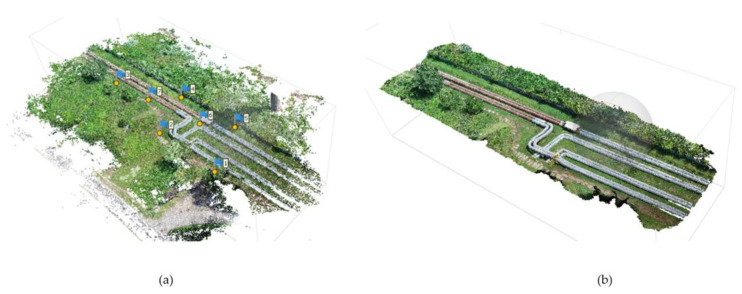
Generated (**a**) sparse point cloud with Ground Control Points and (**b**) dense point cloud.

In this study, the SfM-MVS (Structure from Motion Multi-View Stereo) algorithm was used, a standard solution for UAV image processing. The development of photogrammetric data included several stages. The first stage of work consisted in automatic matching of the photos using the Structure from Motion method based on automatic feature detection between images. As a result of merging the images, a sparse point cloud was obtained (Figure 8a). The next step was to remove the erroneous points twice (approx. 10%) by means of filtration. The first filtration was performed according to the “Reprojection error” criterion, and then “Reconstruction uncertainty”, consisting in finding points that had a low precision of intersection of the homonymous rays. The number of points after filtering the sparse point cloud was 706,549. In the next step, adjustments were carried out using the bundle adjustment method with simultaneous self-calibration and georeferencing. The adjustment process used approximate external orientation parameters of the images. In addition, four GCPs were used, which were located in the corners of the area. The accuracy analysis consisted of determining the RMSE values calculated for the XYZ residuals. The error was 1.6 cm for three check points not included in the alignment.

In the next step, a dense point cloud was generated using the Semi-Global Matching algorithm based on a photogrammetric forward intersection performed for each pixel based on an optimised parallax map. In order to limit the creation time, the range in which the process is to be performed was defined. After this operation, a dense cloud containing 60,354,068 points was obtained (Figure 8b) and the distance between the points was in the range between 10 mm and 50 mm. For further analysis, the dense point cloud was exported to the LAS format, and then it was manually cleaned of noise along the pipes. All the operations described above were performed in the Agisoft Metashape software v1.7.

**Figure 9:**
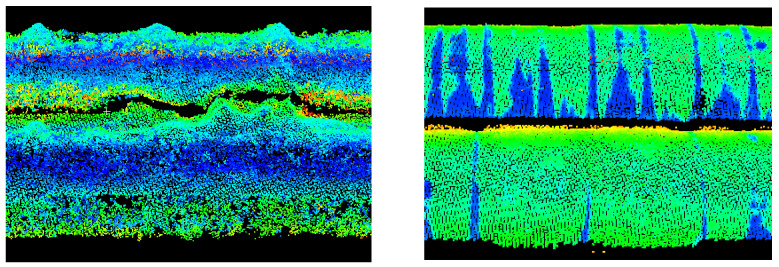
Fragments of the pipeline measured using the structure from motion method: **left**—new pipes (shiny), **right**—old pipes (matte).

Figure 9 illustrates examples of new and old pipeline sections measured using the Structure from Motion method. The point cloud for new pipes with a shiny surface is very deformed. The point cloud for old, rusty and therefore matte pipes shows the correct shape of the object.

## 5. Registration of Point Clouds from All Measurement Methods

Point clouds from different measurement methods occupy different places in space. This is due to the accuracy of the on-board systems of aerial methods. In particular, this applies to the vertical location of point clouds. The aim of the research is to compare the geometric shape of pipelines, measured with each of these methods, in relation to TLS. In order to be able to compare the point clouds obtained by aerial methods with the terrestrial model, it is necessary to shift them appropriately to ensure the most accurate coverage with the TLS cloud. This can be done by integrating aerial clouds with the terrestrial cloud by performing cloud-to-cloud registration to the terrestrial cloud system. In previous studies [38,57], the authors proved that UAV measurements, both photogrammetric and laser scanning ones, can be integrated with the terrestrial cloud in good conditions with an average registration error of a dozen millimetres. Similar steps have been taken in current studies.

The registration was carried out using the Trimble Realworks software, merging each of the aerial clouds to the terrestrial cloud independently. Point clouds for each measurement included pipelines and vegetation (mostly low). The TLS, ULS and SfM measurements were carried out with a mutual time shift of about 2 weeks, which meant that the low vegetation had a different height during each measurement. Therefore, before registration, it was necessary to thoroughly clean the point clouds of the greenery. The smallest average registration error was obtained for the ULS cloud, and it was 21 mm. The registration error for ALS measurements was 28 mm. The registration of SfM measurements turned out to be problematic. When comparing the point clouds measured with this method for old and new pipes (Figure 9), it could be seen that for new, shiny pipes, characteristic saddles were formed between the joints. They were located under the actual surface of the pipelines. Because there were more points on the saddles than on the ridges, the ICP algorithm, when trying to merge such a cloud to the TLS cloud, caused it to tilt. The tilt then affected the old pipes, causing them to be inclined relative to the TLS measurement as well. It was therefore decided to register the SfM clouds with the TLS cloud separately for new and old pipes. As a result, the average registration error for the old pipes was 18 mm, while for the new ones it was larger and reached 27 mm. As a result of the registration, the point clouds from the airborne methods were as close as possible to the surface of the pipes from the TLS method. This made it possible to determine the precision of each method in relation to the TLS measurement.

## 6. Preparation of Models for Analysis

**Figure 10:**
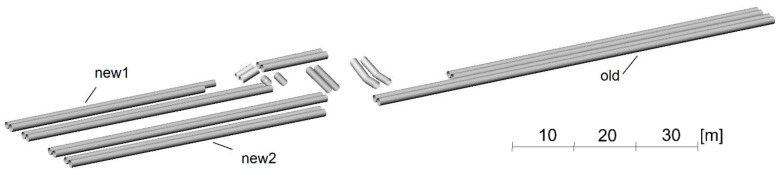
Rectilinear fixed-diameter pipeline segments selected for further analysis. Perspective view. New1, new2 and old—pipes for which additional deviation analyses were performed in the side view.

**Figure 11:**
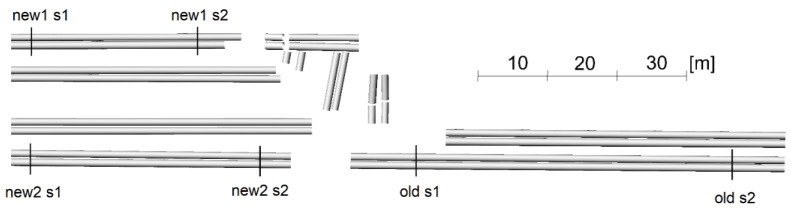
Rectilinear fixed-diameter pipeline segments selected for further analysis. Top view. New1 s1, new1 s2, new2 s1, new2 s2, old s1, old s2—cross-sections marked for which additional deviation analyses were performed.

The research consisted in determining the differences between the reference TLS and aerial measurements. For this purpose, pipeline models were created based on the terrestrial cloud, with respect to which deviations were then determined for each cloud. These models were cylinders fitted into rectilinear parts of the pipelines by the method of least squares in sections where their diameters were constant. Deviations are shown as deviation charts that show the location of deviations and their values, which are grouped into different color-coded ranges. Short curved and diameter-changing fragments were not reconstructed because surface approximation methods that allow modelling of such fragments (mesh or spline) would have contributed additional errors to the analyses. Due to the difference in the material type affecting the accuracy of the results, the tests were carried out in two variants: new and old pipes. Figure 10 and Figure 11 illustrate the created pipe models for which deviation analyses were carried out. The analyses concerned all the pipes in the top view, and then new1, new2 and old pipes in the side view. In addition, side sections were created in the places marked in Figure 11.

## 7. Analysis of Results

Cylindrical models fitted into a point cloud are ideal solids, while point clouds contain local deformations of the object. Therefore, even for terrestrial laser scanning, based on which the models were created, there will be deviations from the model. They demonstrate where pipeline deformations occur. They should be the same for each measurement method. Differences in the results compared to the reference TLS method will identify the suitability of individual aerial methods for determining the geometry of pipelines.

**Figure 12:**
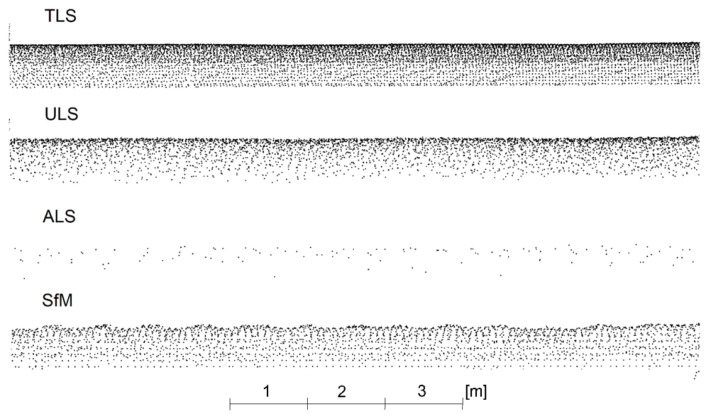
Side view of a fragment of point clouds for old pipes.

**Figure 13:**
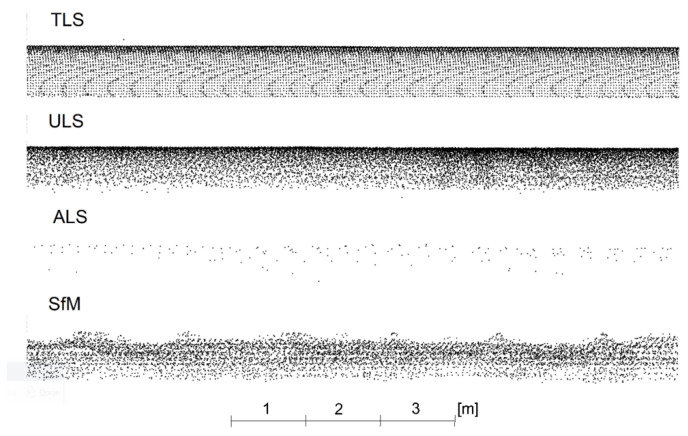
Side view of a fragment of point clouds for new pipes.

The lower parts of the pipes near the ground were cut off from the point clouds intended for analysis. This was due to the different ranges of point coverage by each method performing the measurement from a different trajectory. In addition, for the lower surfaces of the pipes, there was often a significant measurement noise associated with the grass. For the upper surfaces of the pipes, the significant measurement noise occurred only for the SfM method for new pipes, and an attempt was made to remove it before the analyses. Deviation analyses were preceded by the presentation of cloud fragments in the side view, which additionally explained the differences in results observed in the deviation charts. Figure 12 illustrates a side view of the old pipes and Figure 13 of the new pipes.

**Figure 14:**
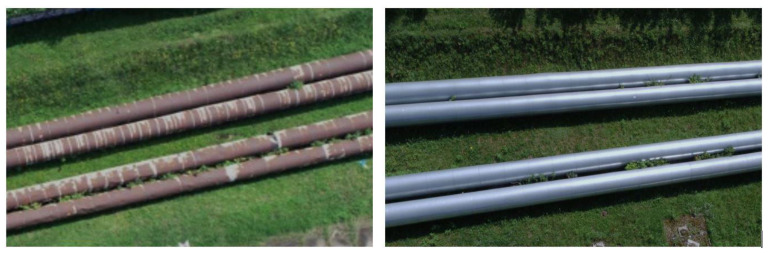
Technical condition of pipelines: old, rusty and deformed (**left**) and new, shiny and undeformed (**right**).

The ULS cloud exhibits the greatest similarity to the TLS cloud. The ALS cloud, due to the low density of points, depicts the side surface of the pipes only to a small extent. The upper surface is characterised by a greater scattering of points compared to the TLS and ULS methods. The SfM cloud shows some undulation of the upper surface of the matte, old pipes and much greater undulation of the shiny, new pipes. They resulted from the different colours and glossiness of the pipe fragments. The old pipes had transverse fragments free of rust but were not shiny (Figure 14 left), and the new pipes had joints between the shiny surfaces (Figure 14, right).

**Figure 15:**
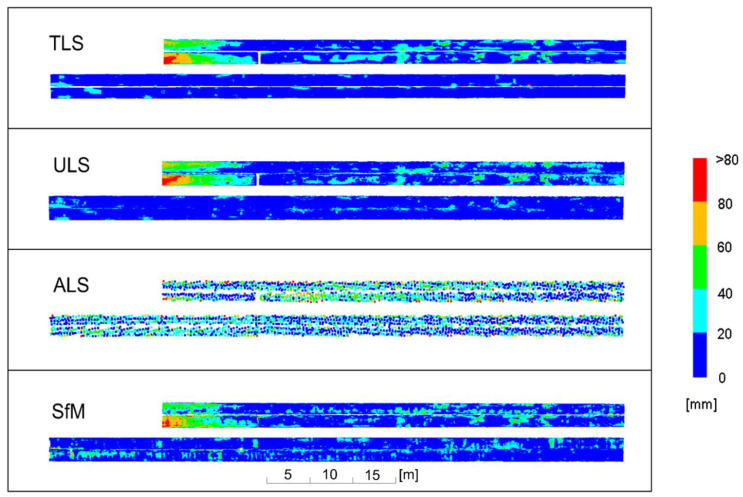
Deviation charts for old pipes with a matte surface, scaled in 20 mm intervals.

**Figure 16:**
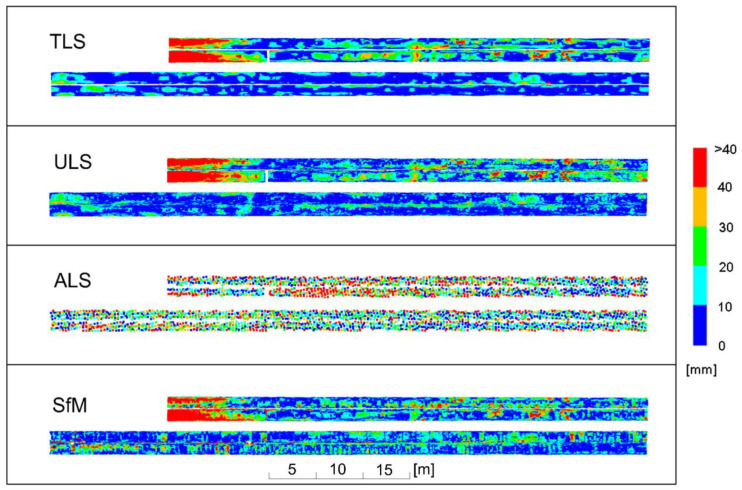
Deviation charts for old pipes with a matte surface, scaled in 10 mm intervals.

Deviation charts were first drawn for old pipes covered with rust, which made them matte. The results are illustrated in Figure 15, grouping deviations in 20 mm intervals, and in Figure 16, grouping deviations in 10 mm intervals.

An analysis of the deviations in 20-mm intervals revealed that the ULS yielded almost the same results as the reference TLS. Deviations from the model occurred in the same places and had almost the same values. Local variations between TLS and ULS fell within a single deviation interval. Similar results were obtained for the SfM method, which, however, for the lower pipes (in the drawing layout) yielded more dispersed deviations differing from TLS by 20 mm. The transverse ribs of the pipes were visible due to the cloud undulations illustrated in Figure 12. Relatively good results were obtained for the ALS cloud, for which the differences in relation to the TLS did not exceed 40 mm for the middle parts of the pipes. Near the edges, the differences were greater and often exceeded 60 mm due to the large size of the laser spot resulting from its incidence at an acute angle on the lateral, inclined surface of the pipes.

When analysing the deviations in 10-mm intervals, it could be observed that the TLS, ULS and SfM methods for the upper pipes (in the drawing layout) generated almost identical results. Isolated deviations with the values exceeding 30 mm occurred almost in the same places for each of the methods. For the lower pipes, the ULS method was compliant with the TLS method within the considered intervals or adjacent intervals (differences up to approximately 10 mm). The SfM measurement exhibited slightly higher values of differences which, however, were scattered on a much larger surface of the pipes. Small islands of deviations occurred where the differences increased to 20–30 mm. The ribs of the pipes resulting from the cloud undulations depicted in Figure 12 became particularly noticeable. The ribs were due to the fact that not the entire surface of the old pipes was covered with rust (Figure 14—left side).

In general, the deformations of the pipelines determined by the reference TLS demonstrated that the pipeline casing had numerous dents. The old pipe covering had traces of repeated human passage through the structure over the years (Figure 14), which caused dents in the casing. In the left part of the upper pipes, the deviation values increased, which was related to the gradual change in the diameter of the pipelines.

**Figure 17:**
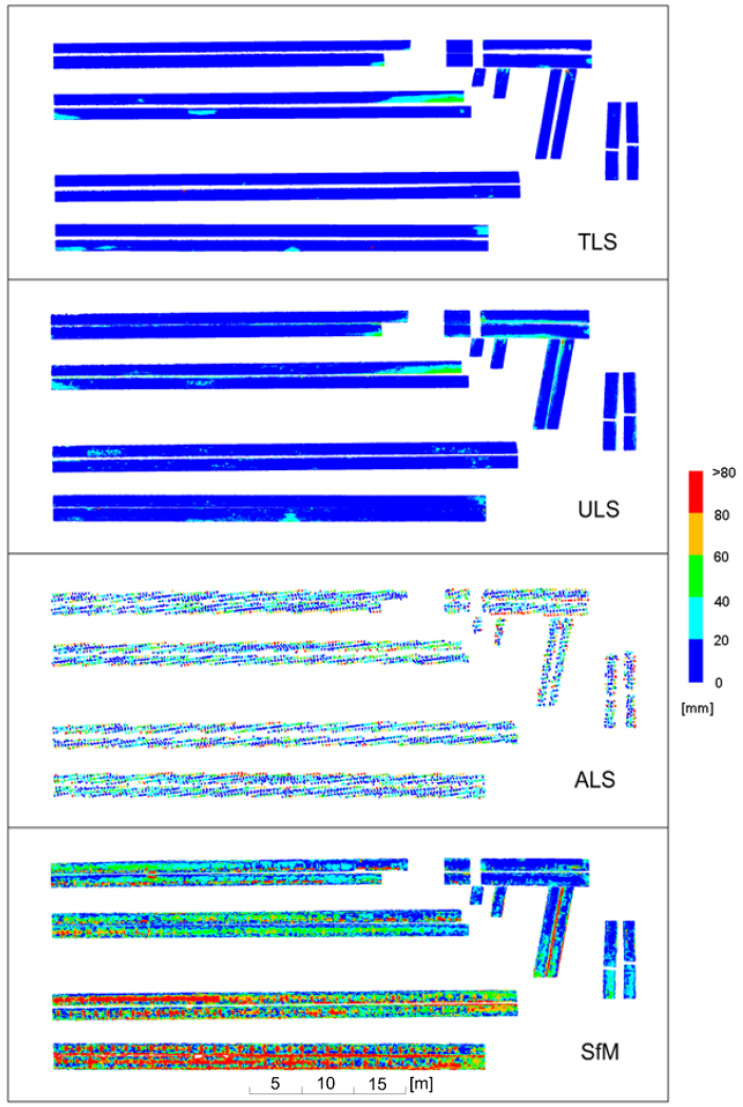
Deviation charts for new pipes with a shiny surface, scaled in 20 mm intervals.

**Figure 18:**
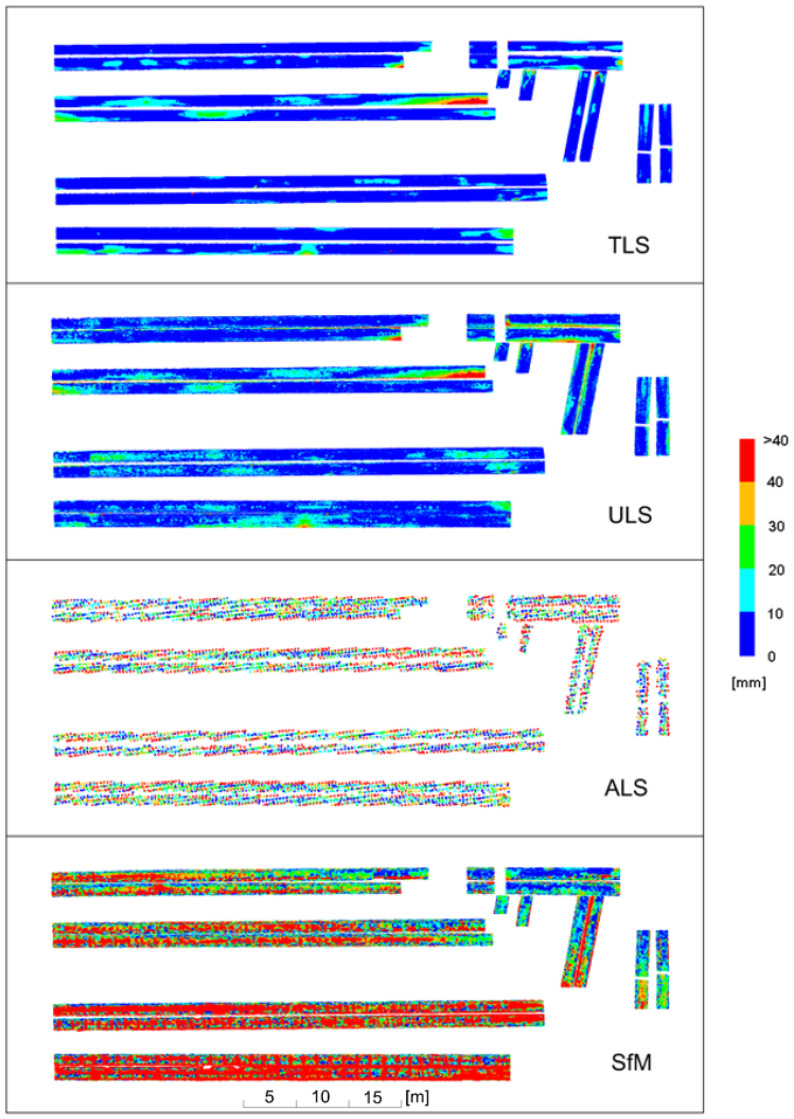
Deviation charts for new pipes with a shiny surface, scaled in 10 mm intervals.

Subsequent analyses were performed for new pipes with a shiny, mostly undeformed surface. The results of the deviation analyses are illustrated in Figure 17, grouping deviations in 20 mm intervals, and in Figure 18, grouping deviations in 10 mm intervals.

The analysis of the deviations in 20 mm intervals confirmed that, similarly to the old pipes, ULS provided practically the same picture of deviations as TLS. Differences of 20 mm occurred in one place in the upper right part of the drawing on the side surface of the pipes. This part of the pipeline was located above the other pipes, and probably one of the UAV flight trajectories had sharp angles of the laser beam incidence on the side, inclined surface of the pipe at this point, which could have resulted in greater measurement errors. ALS, similar to the case of the old pipes, demonstrated differences in deviations compared to the TLS method, reaching up to 40 mm for the central part of the pipes. At their edges, the differences in deviations randomly exceeded 60 mm. Therefore, all the scanning measurements produced a stable image of deviations both for old and new pipes. However, there were significant differences for the SfM method. Deviations for the lower pipes exceeded 80 mm in numerous places. For the upper pipes, they were about 20 mm smaller, and relatively small deviations were obtained for the upper right part of the pipelines. Large deviations resulted mainly from the undulations of the point cloud (Figure 12), associated with reflections from the shiny surface of the pipes. Reflections affected the accuracy of the resulting cloud in different ways, and the deformation values depended on the camera position and lighting conditions for the new pipes.

The analysis of deviations in 10 mm intervals was to determine the differences in relation to the TLS method for more precise applications. Due to the large differences obtained for the SfM method, this analysis was mainly concerned with illustrating the differences between the TLS and ULS methods. Deviations for the ULS method were in most cases consistent with those of the TLS method, although for the lower pipes there were areas where the differences reached about 10 mm. Larger deviations appeared only on the previously discussed short sections of the pipes in the upper right part. Thus, the images of deformations obtained from both methods were similar.

The new pipelines had fewer local deformations than the old pipelines due to a much shorter service life and relatively few dents caused by human passage through the pipelines.

**Figure 19:**
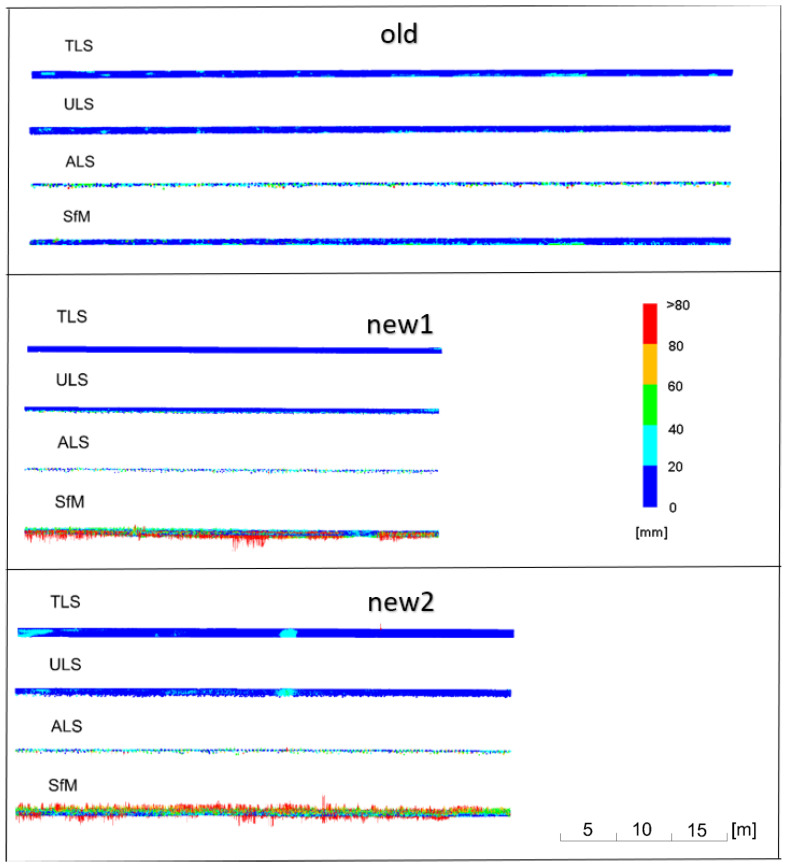
Deviation plots in side view, scaled in 20 mm intervals. Deviation scale 5:1.

The deviation charts in the top views are supplemented with additional deviation charts in the side view, made for several representative pipes marked in Figure 10 as new1, new2 and old. The results of the deviation analyses are demonstrated in Figure 19, grouping deviations in 20 mm intervals. Deviations in the side charts are presented in a 5:1 scale.

**Figure 20:**
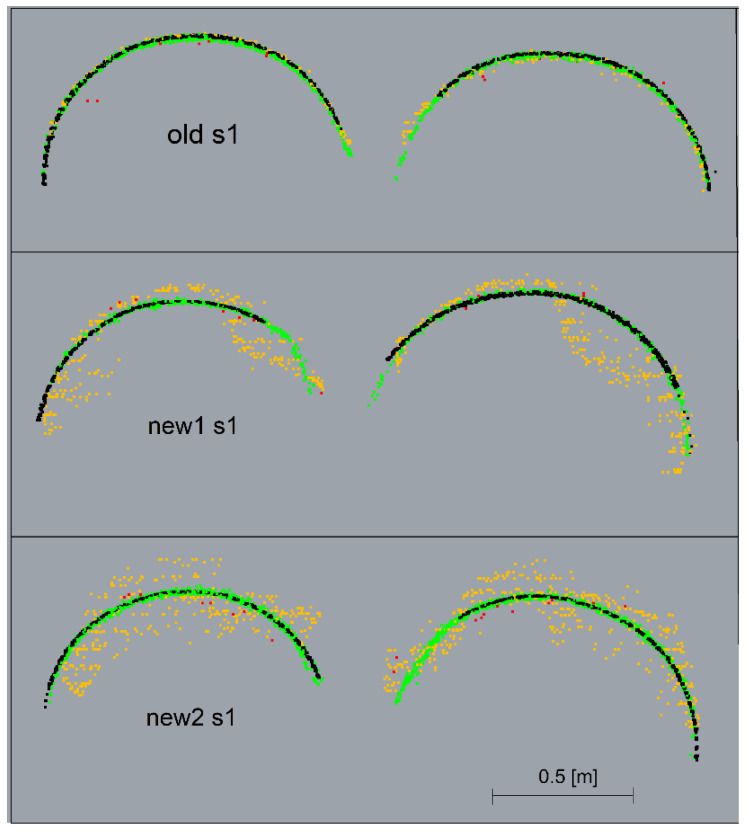
Cross-sections made for left ends of the pipes: old, new1, new2 and pipes paired with them. TLS—black, ULS—green, ALS—red, SfM—orange.

**Figure 21:**
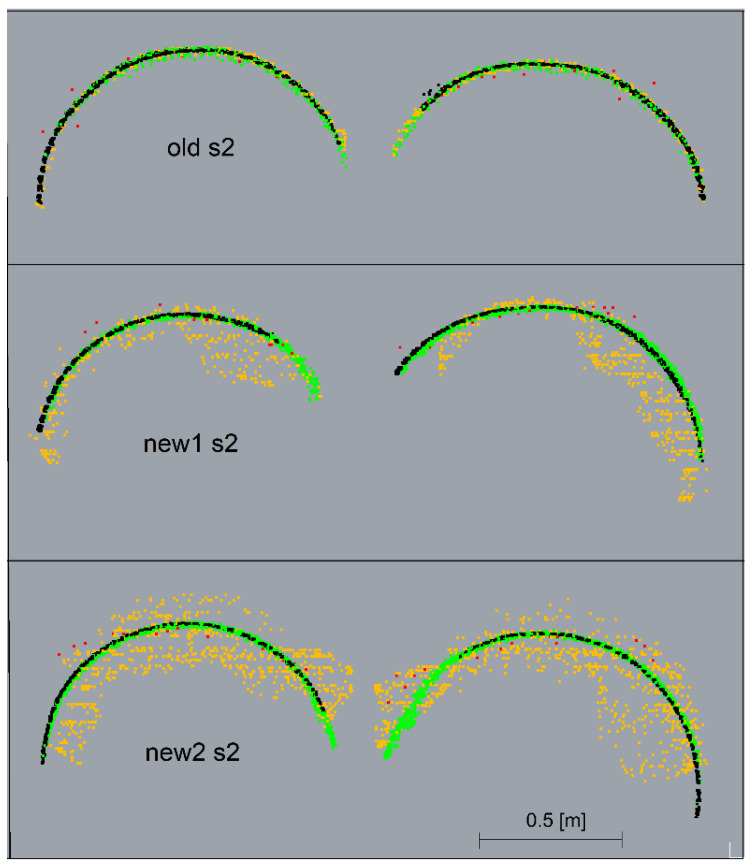
Cross-sections made for right ends of the pipes: old, new1, new2 and pipes paired with them. TLS—black, ULS—green, ALS—red, SfM—orange.

The graphs present a small lateral coverage with ALS measurements, which is due to the low density of the cloud. The TLS, ULS and SfM methods yielded similar results for old matte pipes. The results of the SfM cloud for shiny new pipes had significant deviations. The deviations illustrated in the horizontal view (Figure 17) were smaller for the upper pipes (in the drawing layout) and higher for the lower ones. The side view (Figure 19) demonstrates, however, that the upper pipes only apparently had smaller deviations. The new1 pipe, corresponding to the upper pipes, also had significant deviations. Because of the enlarged scale of the deviations, it is noticeable that they were located mainly on the inside of the pipe and, as a consequence, they were obscured by the deviations above them. The new2 pipe, which corresponded to the lower pipes, had significant deviations both on the outside and inside of the pipe. However, the point cloud on the pipe should form a thin-walled model, so there should be no overlapping of deviations. In order to check what caused the obscuring of deviations, cross-sections of point clouds were made in places marked in Figure 11. Cross-sections were made for both ends of the pipes: old, new1, new2 and the pipes paired with them, and they are presented in Figure 20 and Figure 21.

The cross-sections demonstrated that all the scanning methods as well as the SfM method for old pipes created the expected thin-walled point clouds representing the actual surface of the pipes. However, for shiny pipes, the SfM method created irregular, spatial point clouds that obscured each other on the deviation charts.

The graphical analyses of deviations were complemented by mean deviations (Table 1) determined for each method for old and new pipes.

For old pipes, the mean deviations of the ULS and SfM methods differed slightly by approximately 2–3 mm from the mean deviation for the reference TLS method, which was 12.5 mm. For ALS, the average deviation was twice as large as for TLS. For the new pipes, the TLS measurement deviation was more than twice as small as for old pipes. It resulted from the much better technical condition of the new pipes, on which dents, unlike on the old pipes, were scarce. The average deviation of the ULS method was more than 1.5 times higher than for TLS. The ALS measurement produced a mean deviation comparable to the results for old pipes. The worst results were obtained for the SfM method, for which the mean deviation was more than 10 times higher than the deviation for TLS. The differences in standard deviations were roughly proportional to the differences in mean deviations for each measurement method and pipe type.

## 8. Conclusions

The conducted research made it possible to assess whether and which aerial methods allowed the determination of the geometric shape of pipelines with a precision similar to terrestrial scanning, adopted as a reference method. The best results were obtained for UAV scanning (ULS). Deviations for all the pipes occurred in the same places and had identical values. The results were mostly consistent with terrestrial scanning within 10-mm intervals, and only sometimes did they differ by values that did not exceed 20 mm. Relatively high precision was achieved by ALS. Deviation differences compared to TLS amounted to approximately 40 mm in the middle part of the pipes, increasing, however, to more than 60 mm at the edges. The results of the SfM method depended heavily on light reflections and the type of material. For old, rusty and therefore matte pipes, the results were very close to TLS. The differences became noticeable when analysing deviations in 10-mm intervals, where deviations formed transverse ribs. The ribbing resulted from the technical condition of the pipes, which had non-rusted places between the patches of rust on the joints of the casing. For new, shiny pipes, the differences in deviations compared to TLS were significant, often exceeding 40 and even 60 mm. Most importantly, however, for the shiny pipes, the SfM method created irregular, spatial point clouds that did not correctly describe the thin-walled structure of the pipelines. The studies demonstrated that for pipeline measurements, the only aerial method that provided reproducible high-precision results comparable to terrestrial scanning was UAV scanning (ULS). The results obtained are consistent with those obtained in other studies focusing on the application of SfM and ULS methods to the measurement of civil structures. Unlike buildings, pipelines have a small, rounded cross-section, and significant lengths are covered with metal sheets, which can be glossy. This last factor especially influenced the variability of the SfM method results, while the ULS method showed stable results. The pipelines were lacking in characteristic points and were surrounded by low vegetation. Compared to the best results obtained with structure surveys, these factors degrade the accuracy of merging point clouds from particular scanning drone trajectories.

From the point of view of the economics of data processing, the time taken to perform the measurements and process the data is important. Terrestrial scanning measurements of the surveyed section took 3 h. UAV scanning measurements took 15 min, and photogrammetric measurements took 20 min. The measured and processed aerial scanning data were taken from the ISOK project database. It took about 60 min to process the terrestrial and UAV scanning data, and about 3 h for the UAV photogrammetric data.

## Figures and Tables

**Table 1:** Mean deviations *Dm* determined for each method for old and new pipes.

		Mean Deviations [mm]		
	Old Pipes		New Pipes	
	*Dm*	*σ_Dm_*	*Dm*	*σ_Dm_*
TLS	12.5	12.2	5.6	5.8
ULS	14.5	13.3	9.2	9.7
ALS	26.0	19.8	29.8	23.9
SfM	15.8	13.9	58.6	47.8

## Data Availability

The data presented in this study: (.las files) are available in ISOK project—https://isok.gov.pl/index.html (accessed on 15 April 2023).
